# Obituary

**Published:** 1879-08

**Authors:** 


### OBITUARY.

Died, in Memphis, of yellow fever, on the 20th of July, Dr. J. C. Harris, Dentist, aged about forty years. He was a native of Xenia, Ohio, and there received his education.

He removed to Memphis about the year 1857 and since that time has been in the active practice of his profession in that city. He stood well with his profession, and was highly esteemed as a citizen.

The family will have the warm sympathy of all who knew the husband and father.

